# Combined serum biomarkers in the noninvasive diagnosis of complicated parapneumonic effusions and empyema

**DOI:** 10.1186/s12890-019-0877-8

**Published:** 2019-06-18

**Authors:** Kuo-An Wu, Chih-Ching Wu, Yu-Ching Liu, Pei-Chun Hsueh, Chia-Yin Chin, Chih-Liang Wang, Chi-Ming Chu, Li-Jane Shih, Chia-Yu Yang

**Affiliations:** 1 Department of Internal Medicine, Taoyuan Armed Forces General Hospital, Taoyuan, Taiwan; 2 School of Medicine, Fu-Jen Catholic University, New Taipei City, Taiwan; 3 Department of Medical Biotechnology and Laboratory Science, College of Medicine, Chang Gung University, Taoyuan, Taiwan; 4 Department of Otolaryngology-Head and Neck Surgery, Chang Gung Memorial Hospital, Linkou, Taoyuan, Taiwan; 5 Molecular Medicine Research Center, Chang Gung University, Taoyuan, Taiwan; 6 Graduate Institute of Biomedical Sciences, College of Medicine, Chang Gung University, Taoyuan, Taiwan; 7 School of Medicine, College of Medicine, Chang Gung University, Taoyuan, Taiwan; 8 Division of Pulmonary Oncology and Interventional Bronchoscopy, Department of Thoracic Medicine, Chang Gung Memorial Hospital, Linkou, Taoyuan, Taiwan; 9 Division of Biomedical Statistics and Informatics, School of Public Health, National Defense Medical Center, Taipei, Taiwan; 10 Department of Medical Laboratory, Taoyuan Armed Forces General Hospital, Taoyuan, Taiwan; 11 Department of Microbiology and Immunology, College of Medicine, Chang Gung University, 259 Wen-Hwa 1st Road, Kwei-Shan, Taoyuan, 33302 Taiwan; 12 Division of Colon and Rectal Surgery, Department of Surgery, Chang Gung Memorial Hospital, Taoyuan, Taiwan

**Keywords:** PPE, Serum biomarker, Calprotectin, NGAL, BPI, AZU1

## Abstract

**Background:**

We previously demonstrated that the pleural levels of proteins (neutrophil gelatinase-associated lipocalin/NGAL, calprotectin, bactericidal permeability-increasing/BPI, azurocidin 1/AZU-1) were valuable markers for identifying complicated PPE (CPPE). Herein, this study was performed to evaluate whether these proteins are useful as serological markers for identifying CPPE and empyema.

**Methods:**

A total of 137 participates were enrolled in this study. The levels of NGAL, calprotectin, BPI and AZU-1 were measured in serum and pleural fluid by enzyme-linked immunosorbent assay. We also characterized the diagnostic values of these markers between different groups.

**Results:**

The serum levels of NGAL, calprotectin, and BPI in PPE patients were significantly higher than those in transudates, noninfectious exudates, and healthy controls. The area under the curve (AUC) values of NGAL, calprotectin, and BPI for distinguishing PPE from transudates or noninfectious exudates were around 0.861 to 0.953. In PPE group, serum NGAL and calprotectin levels were significantly elevated in patients with CPPE and empyema than in those with UPPE, whereas the serum BPI levels were similar between these two groups. In CPPE and empyema patients, the serum NGAL showed a positive correlation with the pleural fluid NGAL (*r* = 0.417, *p* <  0.01). When combined with serum CRP, the sensitivity and specificity of serum calprotectin for identifying CPPE and empyema were the highest at 73.52% and 80.55%, respectively.

**Conclusions:**

We concluded that serum calprotectin and NGAL were adjuvant serological markers for CPPE and empyema diagnosis. Patients present with pneumonia and pleural effusion signs in the chest x-ray and the combination of serum calprotectin and CRP constitutes a more highly sensitive and specific assay for identifying CPPE and empyema.

**Electronic supplementary material:**

The online version of this article (10.1186/s12890-019-0877-8) contains supplementary material, which is available to authorized users.

## Background

Globally, pneumonia remains a major cause of morbidity and mortality in lung disease. Parapneumonic effusion (PPE) refers to the filling of the pleural cavity with exudative pleural fluids, and this condition develops secondary to pneumonia [1]. The presence of pleural effusion can be defined with a chest radiograph and/or ultrasound-guided thoracentesis [2]. Identifying the cause of PPE is relatively difficult because patients have the same clinical symptoms, such as cough, chest pain and fever [3]. Traditionally, PPEs are divided into uncomplicated PPE (UPPE), complicated PPE (CPPE), and empyema based on pleural fluid biochemical parameters. Including pH, glucose, and LDH [4, 5]. In addition to routine antibiotic treatments for PPE, patients with severe infection may need an invasive procedure, such as surgery, for resolution [3]. Many reports have indicated that routine and optional pleural fluid tests, including biochemical parameters, proinflammatory cytokines, or enzymes, can be used to guide the clinician toward identifying infectious versus non-infectious pleural effusion and to further stage the PPE [6–8].

Serum biomarkers are feasibly used as a complementary strategy to imaging and histopathological techniques, and the aim of their use is to provide noninvasive and differential information. The acute-phase protein (C-reactive protein [CRP]) and many inflammatory cytokines (IL-6, IL-8, IL-1α, IL-1β and TNF-α) have been studied in both the sera and pleural effusions of pneumonia patients [9–13]. CRP is an acute-phase protein, and the CRP level is widely used as a marker of infectious disease and sepsis [14, 15]. Bielsa et al reported that serum CRP (at the optimum cutoff value of 200 mg/l) had a sensitivity and specificity for identifying CPPE of 58 and 81%, respectively [12]. The pleural levels of various inflammatory cytokines are useful for identifying PPE and CPPE, but the serum levels of these markers are only useful for identifying PPE from other exudates [10, 11, 13]. Additionally, none of the useful markers can predict which PPE patient may need invasive treatment. We previously found that four neutrophil-associated proteins (neutrophil gelatinase-associated lipocalin [NGAL], calprotectin, bactericidal permeability-increasing [BPI] and AZU1) in pleural effusions were significantly elevated in PPE and served as useful markers for identifying CPPE and empyema [16]. The serum levels of these proteins in PPE and noninfectious pleural effusions have not been studied. In this study, we investigate whether the serum levels of these four proteins had diagnostic value and to determine the best combination of serum biomarkers for a rapid and accurate diagnosis of CPPE and empyema.

## Methods

### Study subjects

In total, 137 participants were included in this retrospective study. All experiments were performed in accordance with the guidelines and regulations of the Institutional Review Board at Tri-Service General Hospital (TSGH), Taiwan. Written informed consent was obtained from all patients and/or their families before the sample collection. Patients were classified into five groups (Table 1) according to the cause of the pleural effusion: transudates (*N* = 11), noninfectious exudates (*N* = 22), UPPE (*N* = 40), CPPE (*N* = 17), and empyemas (*N* = 21). A PPE was categorized as UPPE (no organisms found in a culture or on a Gram stain), CPPE (LDH >  1000 units/L; glucose < 60 mg/dL; or pH < 7.2), or empyema (frank pus) [4, 5]. Also, 26 healthy controls were enrolled. After collection, pleural effusions were processed as previously reported. The whole peripheral blood was collected and the blood was clotted at room temperature for 15 min and then centrifuged at 3000 rpm for 10 min. The serum was collected and stored at − 80 °C.

**Table 1:** Clinical characteristics of the healthy controls and patients in this study

Characteristics	Healthy (*N* = 26)	Transudates (*N* = 11)	Noninfectious exudates (*N* = 22)	UPPE (*N* = 40)	CPPE (*N* = 17)	Empyema (*N* = 21)	*p* value^a^
Gender (M; F)	19; 7	10; 1	13; 9	34; 6	15; 2	15; 6	―
Age (years)	68.7; 67 (60–76)	74.7; 83 (59–88)	72.1; 80 (50–88)	71.6; 77 (58–86)	63.4; 58 (50–79)	68.6; 68 (59–82)	0.301
Pleural effusion							
Proteins (g/dl)	―	1.89; 1.86 (1.41–2.20)	3.79; 3.95 (2.91–4.50)	3.83; 3.64 (3.06–4.64)	4.52; 4.56 (4.06–5.08)	3.91; 4.38 (2.80–4.98)	< 0.001
Glucose (mg/dl)	―	173.7; 156.0 (126.0–211.0)	154.2; 147.0 (102.5–178.3)	159.3; 141.0 (112.5–178.5)	47.6; 37.0 (9.0–70.5)	42.0; 21.0 (1.0–51.5)	< 0.001
LDH (U/l)	―	80.3; 81.0 (53.0–95.0)	257.8; 209.0 (134.8–364.8)	360.2; 238.5 (142.8–654.5)	1217.0; 1112.0 (808.5–1546)	7213.0; 2738.0 (956.5–6590)	< 0.001
pH	―	7.465; 7.451 (7.427–7.503)	7.435; 7.426 (7.364–7.480)	7.429; 7.404 (7.335–7.507)	7.192; 7.165 (7.131–7.277)	6.959; 6.957 (6.790–7.204)	< 0.001

Data are presented as the mean; median (25–75 percentile)

^a^The *p* value of the Kruskal-Wallis test presents the difference between these groups

### Measurement of serum and pleural proteins by ELISA

Commercial sandwich ELISA kits were used to detect the serum and pleural effusion levels of BPI (LSBio, WA, USA), NGAL (R&D Systems, MN, USA), azurocidin (AZU1; Abnova, CA, USA), and calprotectin (R&D Systems, MN, USA). Serum CRP was measured with the Beckman Coulter CRP Latex kit (Beckman Coulter, USA). The assays were performed according to a previous study and the manufacturer’s guidelines.

### Statistical analyses

Between-group comparisons were performed with a nonparametric Mann-Whitney U test and Kruskal-Wallis test for two groups and three groups, respectively. The ability of the protein markers to distinguish between CPPE and UPPE or PPE and healthy controls was evaluated using a receiver-operating characteristic (ROC) analysis. ROC curves were generated to illustrate the decision values of various cutoff points for individual proteins in serum or pleural effusions. The point with the largest sum of specificity and sensitivity was selected as the threshold. Spearman correlation was used to measure the associations between serum proteins and pleural effusion proteins. The likelihood ratios for a positive result (LR+) and a negative result (LR-) were calculated with the sensitivity and specificity. All data were processed using SPSS software version 12.0 (SPSS Inc., Chicago, IL, USA). In all analyses, a *p* value < 0.05 was considered to be statistically significant.

## Results

### Clinical characteristics of the study population

A total of 137 participants including healthy controls (*N* = 26), transudates (N = 11), noninfectious exudates (*N* = 22), UPPE (N = 40), CPPE (N = 17), and empyemas (N = 21) were included in this study. The demographic data and pleural levels of the biochemical parameters are shown in Table 1. PE culture was performed on UPPE, CPPE, and empyema cases, and was positive in 0 (0%) of 40 UPPE, 0 (0%) of 17 CPPE, 16 (76.2%) of 21 empyemas. A total of 16 germs were isolated; gram-negative bacteria and gram-positive bacteria were accounted for 56.25% and 37.5%, respectively (Additional file 1: Table S1).

### Elevated serum levels of NGAL, calprotectin, and BPI in PPE patients compared with noninfectious patients and healthy controls

The levels of NGAL, calprotectin, BPI, and AZU1 were analyzed by sandwich ELISA. The serum levels of NGAL, calprotectin, and BPI in PPE patients (UPPE, CPPE, and empyema; *N* = 78) were significantly higher than those in transudates, noninfectious exudates, and healthy controls (Additional file 1: Figure S1 and Table 2). The serum levels of NGAL, calprotectin, and BPI in PPE patients were estimated (expressed as the mean value ± s.e.m.) as 260.90 ± 23.84 ng/ml, 6.80 ± 0.63 μg/ml, and 142.52 ± 8.03 pg/ml, respectively (Table 2). The area under the curve (AUC) values for distinguishing PPE from transudates were 0.953 for serum NGAL, 0.907 for serum calprotectin, and 0.904 for serum BPI (Table 3). The area under the curve (AUC) values for distinguishing PPE from noninfectious exudates were 0.947 for serum NGAL, 0.905 for serum calprotectin, and 0.861 for serum BPI (Table 3). Of the three proteins, serum NGAL had the best diagnostic value for PPE from transudates or noninfectious exudates. However, the serum levels of AZU1 were similar between PPE, transudates, noninfectious exudates and healthy control groups (Table 2). In our cohorts, serum IL-6 and CRP was also significantly increased in PPE patients compared with transudates, noninfectious exudates, and healthy controls (Table 2).

**Table 2:** The levels of serum biomarkers in healthy controls and patients

Serum	Healthy (*N* = 26)	Transudates (*N* = 11)	Noninfectious exudates (*N* = 22)	UPPE (*N* = 40)	CPPE (*N* = 17)	Empyema (*N* = 21)	PPE (*N* = 78)
NGAL (ng/ml)	47.8; 44.7 (35.6–59.0)	53.4; 49.3 (34.5–60.1)	53.0; 52.8 (33.9–68.1)	165.0; 148.4 (88.1–219.6)	322.0; 271.2 (187.6–443.4)	394.1; 330.3 (184.1–600.7)	260.9; 206.7 (117.5–322.0)
Calprotectin (ug/ml)	1.3; 1.3 (1.0–1.7)	1.6; 1.5 (1.1–1.8)	1.6; 1.6 (1.1–1.9)	4.6; 3.5 (1.8–5.9)	8.6; 6.7 (4.5–11.9)	9.5; 7.1 (5.1–12.2)	6.8; 5.3 (2.8–8.5)
BPI (pg/ml)	23.8; 21.5 (8.9–33.2)	40.1; 41.4 (7.8–61.7)	50.7; 40.0 (7.8–81.5)	134.3; 140.9 (103.7–167.2)	145.5; 137.4 (96.0–169.7)	155.8; 144.2 (98.1–207.3)	142.5; 141.2 (101.3–169.2)
AZU1 (ng/ml)	14.3; 13.7 (5.1–20.4)	16.6; 11.5 (8.5–24.6)	14.8; 13.1 (5.3–21.8)	16.1; 15.6 (9.9–19.8)	18.9; 16.5 (12.3–23.4)	15.9; 16.8 (8.7–20.0)	16.7; 15.7 (9.9–20.2)
IL-6 (pg/ml)	5.3; 2.0 (0.4–3.7)	13.4; 10.5 (0.4–21.3)	69.8; 20.1 (4.0–53.9)	70.0; 29.6 (13.1–99.3)	79.1; 51.1 (29.0–118.0)	223.5; 161.2 (40.0–376.5)	113.3; 43.1 (20.5–138.2)
CRP (mg/dl)^a^	0.3; 0.2 (0.1–0.4)	5.3; 4.4 (1.3–10.3)	6.3; 5.6 (1.5–11.2)	12.4; 10.6 (7.5–17.6)	18.4; 18.2 (13.5–22.5)	25.4; 27.8 (16.9–35.0)	17.4; 15.9 (9.8–24.1)

Data are presented as the mean; median (25–75 percentile)

*PPE* parapneumonic effusion is refer to UPPE, CPPE, and empyema. *NGAL* Neutrophil gelatinase-associated lipocalin; *BPI* Bactericidal permeability-increasing protein; *AZU1* Azurocidin. ^a^The CRP data are missing for eight patients

**Table 3:** The diagnostic accuracy of the serum biomarkers for distinguishing PPE from transudates or noninfectious exudates

Serum	Cutoff	Sensitivity (%)	Specificity (%)	AUC (95% confidence interval)
PPE vs. transudates				
NGAL	> 101.9 ng/ml	82.1	100.0	0.953 (0.906–1.000)
Calprotectin	> 2.6 μg/ml	78.2	100.0	0.907 (0.842–0.971)
BPI	> 88.5 pg/ml	80.8	100.0	0.904 (0.842–0.967)
CRP	> 7.9 mg/dl	81.6	72.7	0.861 (0.765–0.957)
IL-6	> 12.6 pg/ml	89.7	72.7	0.865 (0.759–0.970)
PPE vs. noninfectious exudates				
NGAL	> 106.9 ng/ml	80.8	100.0	0.947 (0.907–0.987)
Calprotectin	> 2.5 μg/ml	79.5	100.0	0.905 (0.848–0.962)
BPI	> 113 pg/ml	73.1	90.9	0.861 (0.780–0.942)
CRP	> 8.5 mg/dl	80.3	72.7	0.831 (0.744–0.918)
IL-6	> 25.4 pg/ml	71.8	63.6	0.685 (0.545–0.825)

*AUC* Area under the ROC curve. *NGAL* Neutrophil gelatinase-associated lipocalin, *BPI* Bactericidal permeability-increasing protein, *CRP* C-reactive protein

### Diagnostic value of serum levels of NGAL and calprotectin for identifying patients with CPPE and empyema

We further investigated the serum levels of these four proteins in CPPE, empyema, and UPPE. The serum levels of NGAL and calprotectin were significantly elevated in CPPE and empyema compared with UPPE (Additional file 1: Figure S1 and Table 2). Otherwise, the serum levels of BPI and AZU1 were similar between CPPE, empyema, and UPPE (Additional file 1: Figure S1 and Table 2). In the CPPE and empyema patients, the serum CRP and serum IL-6 levels were significantly elevated, and these results were consistent with those of previous reports (Table 2). The AUC values for distinguishing CPPE and empyema from UPPE were 0.775 for serum NGAL, 0.765 for serum calprotectin, and 0.766 for serum CRP, and 0.717 for serum IL-6 (Table 4). The diagnostic value of the individual serum biomarker was similar.

**Table 4:** Diagnostic accuracy of individual serum and pleural fluid tests for distinguishing CPPE and empyema from UPPE

	Cutoff	Sensitivity (%)	Specificity (%)	LR+	LR-	AUC (95% confidence interval)
Serum						
NGAL (ng/ml)	> 220 ng/ml	68.4	77.5	3.04	0.41	0.775 (0.672–0.878)
Calprotectin (μg/ml)	> 6 μg/ml	65.8	77.5	2.92	0.44	0.765 (0.661–0.870)
CRP (mg/dl)	> 12.5 mg/dl	85.3	69.4	2.79	0.21	0.766 (0.651–0.881)
IL-6 (pg/ml)	> 31 pg/ml	82.9	55.3	1.85	0.31	0.717 (0.601–0.833)
Pleural effusion						
NGAL (ng/ml)	> 600 ng/ml	78.9	85.0	5.26	0.25	0.855 (0.770–0.941)
Calprotectin (μg/ml)	> 90 μg/ml	84.2	97.5	33.68	0.16	0.965 (0.929–1.000)
BPI (ng/ml)	> 10 ng/ml	97.4	87.5	7.79	0.03	0.970 (0.940–1.000)
AZU1 (ng/ml)	> 175 ng/ml	97.4	57.5	2.29	0.05	0.860 (0.781–0.939)
CRP (mg/dl)	> 7.3 mg/dl	84.2	87.2	6.58	0.18	0.879 (0.800–0.957)
IL-6 (ng/ml)	> 31 ng/ml	81.6	72.5	2.97	0.25	0.818 (0.719–0.918)

*LR+* Positive likelihood ratio, *LR*- Negative likelihood ratio, *AUC* Area under the ROC curve, *NGAL* Neutrophil gelatinase-associated lipocalin, *BPI* Bactericidal permeability-increasing protein, *AZU1* Azurocidin, *CRP* C-reactive protein

We also evaluated the pleural levels of these four proteins in patients with PPE. The pleural levels of these four proteins were all significantly elevated in CPPE and empyema compared with UPPE (Additional file 1: Table S2). The AUC values for distinguishing CPPE and empyema from UPPE were from 0.855 to 0.970 for these four pleural biomarkers (Table 4). These findings were consistent with those of our previously reports. The diagnostic values of the pleural NGAL and calprotectin were similar. The levels of CRP and IL-6 were significantly higher in pleural fluid from patients with CPPE and empyema. Furthermore, the analysis of the pleural to serum ratios of the NGAL and calprotectin proteins showed significant differences in CPPE, empyema, and UPPE (Additional file 1: Table S3). Compared with serum levels of NGAL, the pleural levels of NGAL were increased by approximately 3.46-fold and 9.4-fold in UPPE and CPPE/empyema, respectively. Compared with the serum levels of calprotectin, the pleural levels of calprotectin were increased by approximately 10.23-fold and 22.60-fold in UPPE and CPPE/empyema, respectively. The pleural to serum ratio of calprotectin had an AUC value of 0.784 for distinguishing CPPE and empyema from UPPE (Additional file 1: Table S3).

Table 5 shows the correlations between these four proteins in the serum and pleural effusions of all patients. The serum levels of NGAL and calprotectin showed a statistically significantly positive correlation with the pleural levels of NGAL (*r* = 0.420) and calprotectin (*r* = 0.433), respectively (Table 5). In the CPPE and empyema patients, the serum levels of NGAL also showed a statistically significantly positive correlation with the pleural levels of NGAL (*r* = 0.417) (Additional file 1: Figure S2).

**Table 5:** Spearman correlation between the pleural fluid and serum levels of the four proteins in PPE patients

Parameters	PE NGAL	PE Calprotectin	PE BPI	PE AZU1	Serum NGAL	Serum Calprotectin	Serum BPI	Serum AZU1
PE NGAL	―	0.594**	0.625^**^	0.708^**^	0.420^**^	0.307^**^	0.054	0.283^*^
PE calprotectin	―	―	0.744^**^	0.626^**^	0.374^**^	0.433^**^	0.058	0.009
PE BPI	―	―	―	0.601^**^	0.429^**^	0.357^**^	0.168	0.085
PE AZU1	―	―	―	―	0.222	0.195	−0.098	0.234^*^
Serum NGAL	―	―	―	―	―	0.373^**^	0.338^**^	0.264^*^
Serum calprotectin	―	―	―	―	―	―	0.327^**^	0.321^**^
Serum BPI	―	―	―	―	―	―	―	0.155
Serum AZU1	―	―	―	―	―	―	―	―

### The combination of serum biomarkers for the noninvasive diagnosis of CPPE and empyema

**Fig. 1:**
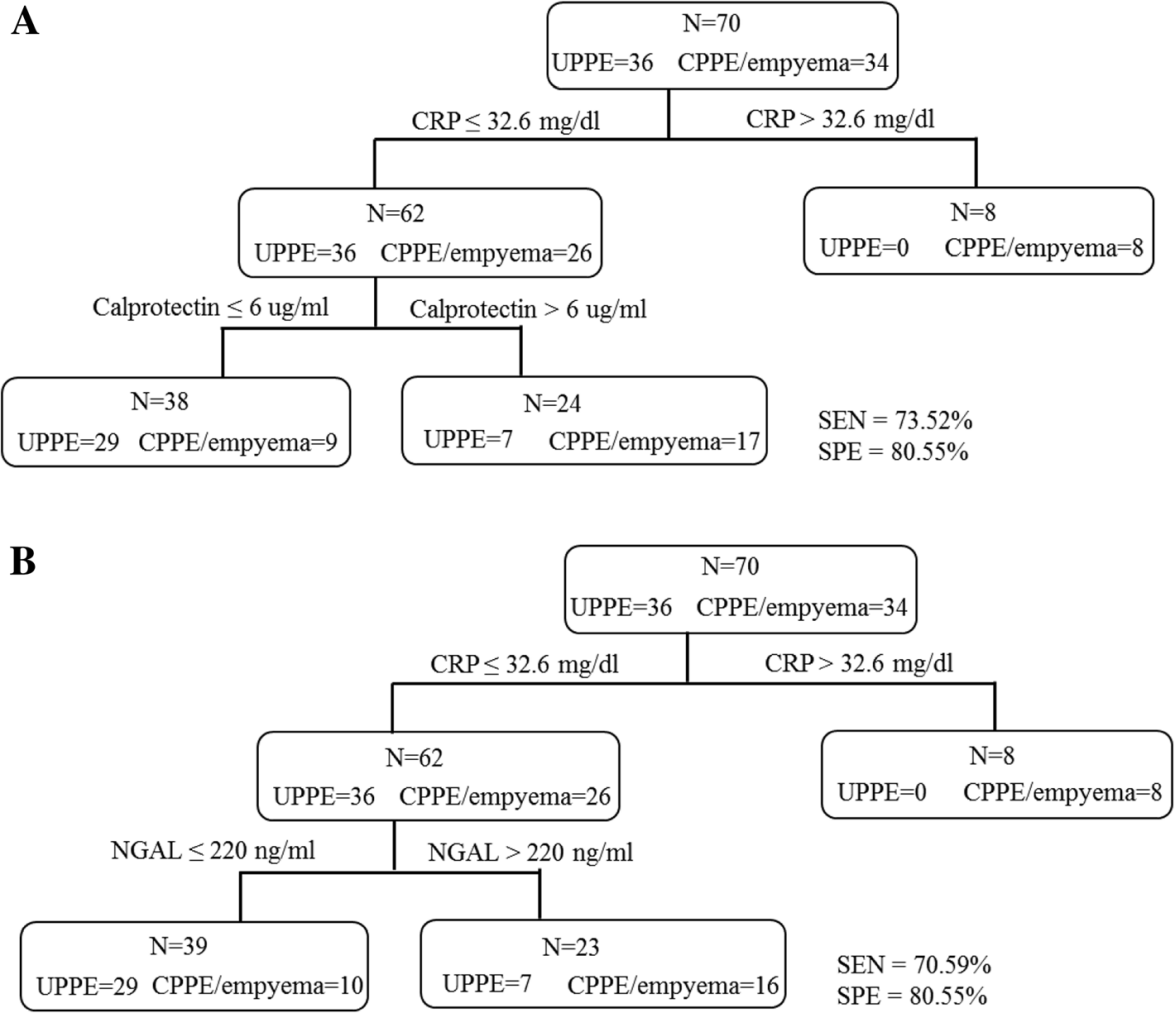
The diagnostic power of serum CRP, calprotectin, and NGAL for identifying CPPE and empyema. Diagnostic power of serum CRP combined with calprotectin (**a**) and serum CRP combined with NGAL (**b**) in CPPE and empyema

According to the ELISA data, we observed that the serum levels of NGAL, calprotectin, and CRP were significantly elevated in CPPE. However, the sensitivity and specificity of a single marker in serum for distinguishing CPPE from UPPE were not efficient for clinical usage. We sought to combine these markers to improve the diagnostic power. We performed a subgroup analysis of patients with serum CRP levels > 32.6 mg/dl, which approach can identify all severe patients. Serum CRP levels above 32.6 mg/dl were evident in 8 cases in the CPPE and empyema group but in no cases in the UPPE group (Fig. 1). The others 62 patients underwent serum calprotectin tests; the optimal cutoff value for calprotectin was 6 μg/ml, and the test was considered positive for calprotectin > 6 μg/ml. According to these criteria, 17 and 9 cases in the CPPE groups were considered positive and negative, respectively. Additionally, 29 and 7 cases in the UPPE groups were considered negative and positive, respectively. Thus, the combination of the serum levels of CRP and calprotectin improved the sensitivity to 73.52% and specificity to 80.55% for identifying CPPE and empyema (Fig. 1a). When we combined the CRP and NGAL as biomarkers for identifying CPPE and empyema, the result was shown in Fig. 1b. The sensitivity and specificity were 70.59 and 80.55%, respectively (Fig. 1b).

## Discussion

The clinical symptoms and chief complaints of UPPE and CPPE are similar and one of the key diagnostic challenges is how to rapidly identify CPPE and empyema patients who require an invasive intervention. In this study, we demonstrated that patients with PPE expressed significantly higher serum levels of NGAL, calprotectin, and BPI than those in transudates, noninfectious exudates, and healthy controls. Furthermore, serum levels of NGAL and calprotectin were significantly elevated in CPPE and empyema compared to UPPE. The combination of serum calprotectin and CRP constitutes a more highly sensitive and specific assay for identifying CPPE and empyema.

Elevated serum NGAL levels have been reported in kidney disease, sepsis, heart disease, metabolic diseases and cancers [17–20]. Additionally, serum NGAL increases with community-acquired pneumonia (CAP) and is an independent predicator of hospital mortality in CAP patients with a cutoff value 457 ng/ml [21]. Gumus et al reported that both serum and pleural NGAL levels are highly effective in differentiating PPE from non-PPE [22]. In sepsis patients, Otto et al analyzed 15 patients with severe sepsis, and the mean concentration of plasma NGAL was approximately 381.7 ng/ml [23]. However, these previous reports did not subgroup the PPE patients into CPPE, empyema, or UPPE categories. In our study, the serum NGAL levels of healthy individuals, transudates, and noninfectious exudates were approximately 47.8 to 53.4 ng/ml. However, the serum levels in UPPE and CPPE/empyema were increased by approximately 3-fold (165.0 ng/ml) and 7-fold (322 to 361 ng/ml), respectively. These results may indicate that circulating neutrophils produce higher NGAL levels under the severe inflammation that is typical of pneumonia patients with CPPE.

Many pathological conditions, such as infection and inflammation, are associated with altered calprotectin levels in body fluids [24–26]. A study by van Zoelen et al. showed that serum calprotectin was elevated in sepsis caused by pneumonia and that the mean concentration of serum calprotectin was 4 μg/ml in 29 pneumonia patients; however, there was no apparent correlation between serum calprotectin and the severity of disease [27]. The calprotectin levels in pleural effusion are significantly lower in malignant PE than in benign effusions and have a high accuracy for predicting malignancy in patients with exudative PE [28]. The diagnostic accuracy of serum calprotectin for predicting CPPE/empyema in patients has not been addressed previously. Our results directly demonstrate that serum calprotectin is elevated in patients with PPE compared to healthy controls, transudates, and noninfectious exudates. Furthermore, serum calprotectin is higher in CPPE and empyema (8.6 to 9.5 μg/ml) than in UPPE (4.6 μg/ml).

C-reactive protein (CRP) is a well-known marker for sepsis and/or infection [14, 15]. Increased serum CRP levels are associated with invasive infections and various diseases [15, 29]. In our study, the AUC value of CRP only in identifying PPE from transudates and noninfectious exudates were 0.861 and 0.831, respectively. The diagnostic value of CRP in identifying PPE from other types of effusion, particularly CPPE/empyema was not higher than those three serum biomarkers (NGAL, calprotectin, BPI). The individual protein of these three serum biomarkers was also not good enough for clinical. When we set the cutoff value of serum CRP was 32.6 mg/dl, the sensitivity was 100% for identifying CPPE/empyema, which approach can identify all severe patients. Patients with serum CRP lower than 32.6 mg/dl may perform second serum test and the combination of neutrophil-associated proteins (calprotectin or NGAL) and CRP could provide useful information for CPPE and empyema diagnosis.

The pleural to serum ratios of the NGAL and calprotectin proteins showed significant differences in CPPE/empyema and UPPE. For NGAL, the pleural/serum ratios were 9.30 and 3.46 in CPPE/empyema and UPPE, respectively. For calprotectin, the pleural/serum ratios were 22.60 and 10.23 in CPPE/empyema and UPPE, respectively. These results indicated that the proteins were most elevated in the local inflammation site compared to the systemic circulation. Furthermore, the pleural/serum ratios of NGAL and calprotectin were higher in CPPE/empyema than in UPPE (*p* = 0.015 for NGAL, *p* <  0.001 for calprotectin). The accuracy of serum calprotectin alone (AUC = 0.765) and the pleural/serum ratio of calprotectin (AUC = 0.786) were similar in confirmed CPPE and empyema.

The main limitation of our study was the small sample size and patients were recruited from only one hospital. Thus, further large-scale studies that enroll various causes of pleural effusion will enable further evaluation of the diagnostic value of these serological markers. The other limitation was the sensitivity and specificity values of serological markers were not high enough and not superiority than pleural markers we had identified. This phenomenon may due to serum is a heterogeneous body fluid sample contained various proteins and may be affected by different physiological conditions. Third, only one time-point specimen was obtained from patients, and we could not evaluate the serial NGAL or calprotectin levels of multiple hospital days. The kinetics of serological markers may be helpful for PPE diagnosis and for monitoring disease severity or therapeutic response. In summary, we concluded that patients present with pneumonia and pleural effusion signs on the chest x-ray and the combination of the serum CRP with the serum calprotectin constitutes a more highly sensitive and specific assay for the differential diagnosis of UPPE and CPPE/empyema. These findings can be helpful in early clinical decision-making for the treatment of these patients as these markers may lead to a better prognosis and the avoidance of potential adverse consequences.

## Additional file

Additional file 1: **Figure S1.** Box plots of serum concentrations of four proteins in healthy controls and patients. **Figure S2.** Correlation between the serum levels and pleural levels of NGAL and calprotectin. **Table S1.** Micorbial isolates in PPE. **Table S2.** Pleural fluid concentrations of proteins in PPE patients. **Table S3** Pleural to serum ratio of NGAL and calprotectin in PPE. (DOCX 668 kb)

## Data Availability

The datasets used and/or analysed during the current study are available from the corresponding author on reasonable request.

## Abbreviations

AZU1: Azurocidin 1
BPI: Bactericidal permeability-increasing
CPPE: Complicated parapneumonic effusions
CRP: C-reactive protein
NGAL: Neutrophil gelatinase-associated lipocalin
PPE: Parapneumonic effusion
UPPE: Uncomplicated parapneumonic effusions
