# Supplementary material for: Combined serum biomarkers in the noninvasive diagnosis of complicated parapneumonic effusions and empyema
Source: BMC Pulm Med. 2019 Jun 18;19:108. doi: 10.1186/s12890-019-0877-8 (PMC6582530; doi:10.1186/s12890-019-0877-8)
Supplement: Supplementary file 1 — Figure S1. Box plots of serum concentrations of four proteins in healthy controls and patients. Figure S2. Correlation between the serum levels and pleural levels of NGAL and calprotectin. Table S1. Micorbial isolates in PPE. Table S2. Pleural fluid concentrations of proteins in PPE patients. Table S3 Pleural to serum ratio of NGAL and calprotectin in PPE. (DOCX 668 kb) [file 12890_2019_877_MOESM1_ESM.docx]

**Supplementary Material**


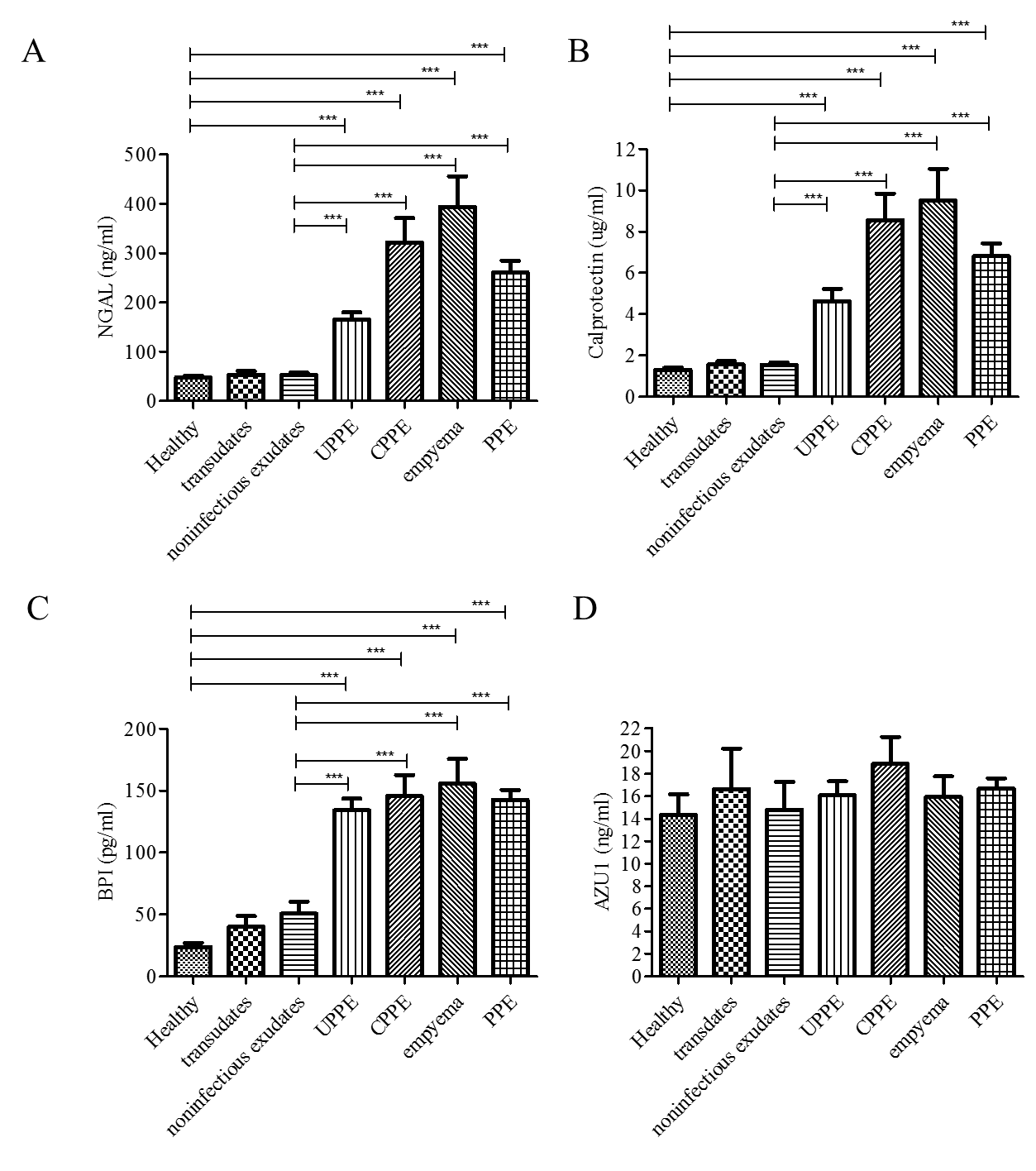
**Figures**

Figure 1. Box plots of serum concentrations of four proteins in healthy controls and patients. Serum levels of (A) NGAL, (B) calprotectin, (C) BPI, (D) AZU1 in different groups were measured by ELISA assay. UPPE: uncomplicated parapneumonic effusion; CPPE: complicated parapneumonic effusion. PPE: parapneumonic effusion is refer to UPPE, CPPE, and empyema. The statistical powers were estimated around 90% to 100%.

A NGAL


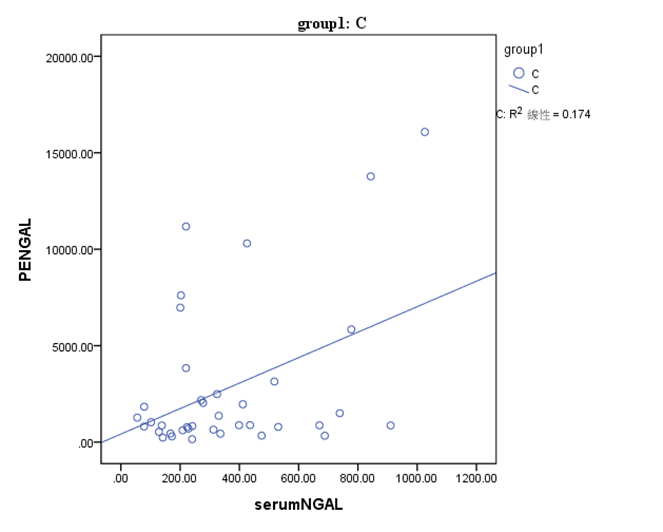


CPPE and empyema (N= 38)

UPPE (N = 40)


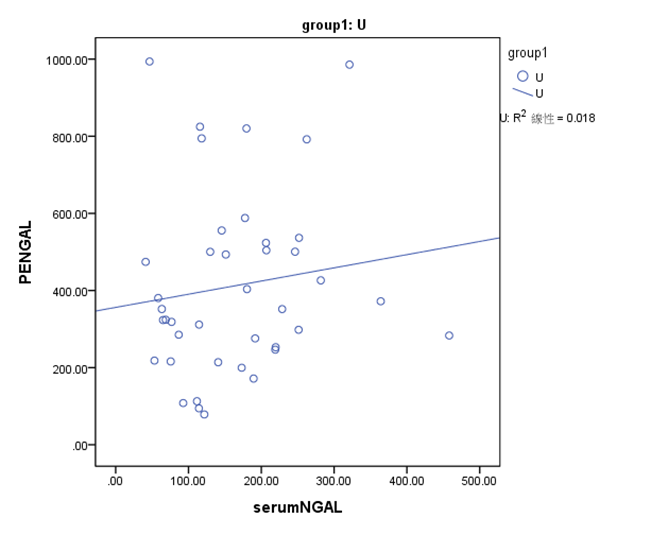


*r =* 0.417

*P =* 0.009

*r =* 0.134

*P =* 0.409

B Calprotectin


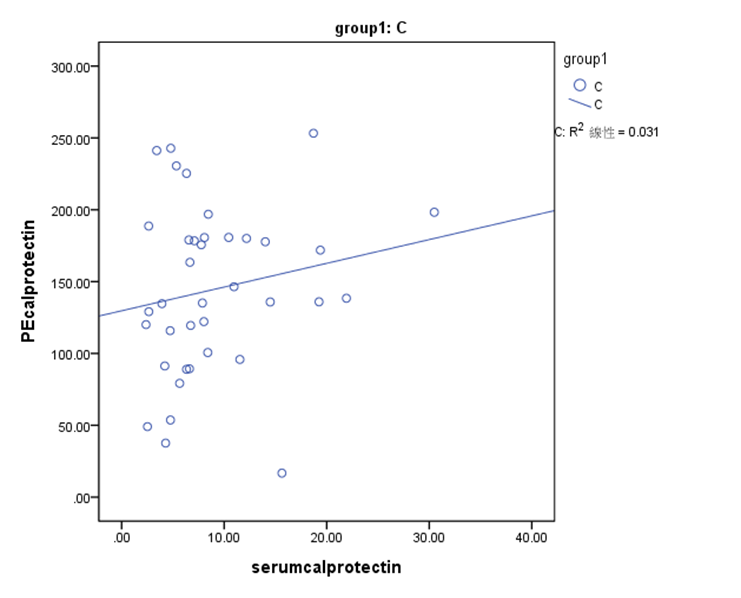

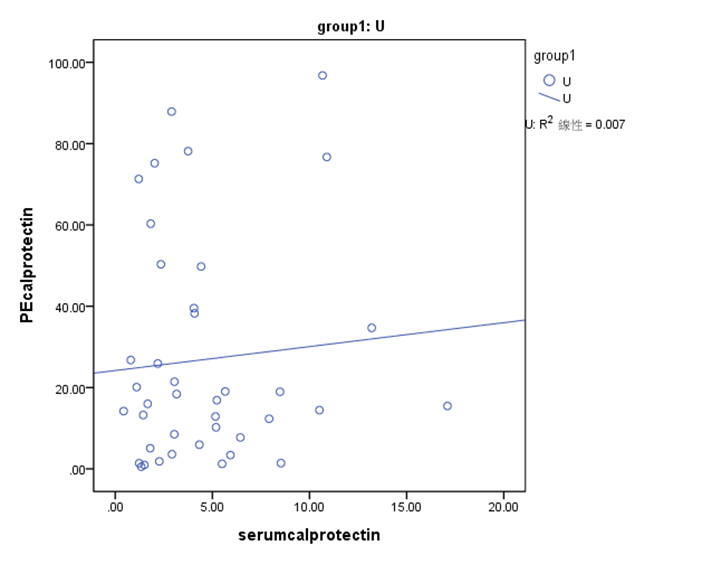


CPPE and empyema (N= 38)

UPPE (N = 40)

*r =* 0.176

*P =* 0.290

*r =* 0.084

*P =* 0.620

Figure 2. Correlation between the serum levels and pleural levels of NGAL and calprotectin.

**Tables**

**Table 1. Micorbial isolates in PPE.**

| pathogens | No.(%) |
| --- | --- |
| Gram-positive bacteria | 6 (37.5%) |
| *Staphylococcus aureus* | 2 |
| *Streptococcus constellatus* | 2 |
| *Actinomyces meyeri* | 1 |
| *Bacilli* | 1 |
| Gram-negative bacteria | 9 (56.25%) |
| *Klebsiella pneumoniae* | 5 |
| *Enterobacter cloacae* | 1 |
| *Escherichia coli* | 1 |
| *Salmonella group D* | 1 |
| *Bacteroides distasonis* | 1 |
| Fugus | 1 (6.25%) |
| *Candida albicans* | 1 |

**Table 2. Pleural fluid concentrations of proteins in PPE patients.**

| Pleural | UPPE (N = 40) | CPPE (N = 17) | Empyema (N = 21) | *p* Value^a^ | *p* Value^b^ |
| --- | --- | --- | --- | --- | --- |
| NGAL (ng/ml) | 412.7; 351.9 (248.1 - 518.7) | 2091.0; 1499.0 (628.2 - 2815.0) | 3387.0; 872.4 (655.6 - 4403.0) | < 0.001 | < 0.001 |
| Calprotectin (ug/ml) | 26.9; 16.4 (6.4 - 39.2) | 115.6; 120.1 (93.5 - 137.1) | 168.3; 179.0 (129.0 - 211.8) | < 0.001 | < 0.001 |
| BPI (pg/ml) | 11.8; 6.8 (4.0 - 8.3) | 248.4; 221.7 (53.2 - 437.4) | 300.3; 253.2 (71.0 - 541.3) | < 0.001 | < 0.001 |
| AZU1 (ng/ml) | 265.5; 128.3 (36.3 - 463.9) | 1183.0; 1441.0 (511.8 - 1787.0) | 981.5; 527.9 (361.3 - 1794.0) | < 0.001 | < 0.001 |
| IL-6 (ng/ml) | 29.0; 15.1 (5.5 - 52.6) | 98.1; 110.9 (60.7 - 130.3) | 75.6; 90.8 (19.8 - 111.2) | < 0.001 | < 0.001 |
| CRP (mg/dl) | 4.7; 4.3 (2.6 - 6.1) | 10.2; 9.1 (8.1 - 12.6) | 11.6; 11.9 (8.0 - 15.3) | < 0.001 | < 0.001 |

Data are presented as the mean; median (25 - 75 percentile)

^a^The p value of the Kruskal-Wallis test presents the difference between these three groups. ^b^ The p value of the Mann-Whitney U test presents the difference between uncomplicated parapneumonic effusion (UPPE) and complicated parapneumonic effusion (CPPE).

**Table 3. Pleural to serum ratio of NGAL and calprotectin in PPE.**

| Pleural/serum ratio | UPPE | CPPE and empyema | *p* value | Cutoff | Sensitivity (%) | Specificity (%) | LR+ | LR- | AUC (95% confidence interval) |
| --- | --- | --- | --- | --- | --- | --- | --- | --- | --- |
| NGAL | 3.46 ± 0.57 | 9.30 ± 1.89 | 0.015 | > 4 | 52.6 | 75 | 2.10 | 0.63 | 0.660 (0.536-0.783) |
| Calprotectin | 10.23 ± 2.11 | 22.60 ± 2.75 | < 0.001 | > 11 | 78.9 | 75 | 3.16 | 0.28 | 0.784 (0.678-0.889) |

LR+: positive likelihood ratio; LR-: negative likelihood ratio; AUC: area under the ROC curve; NGAL: neutrophil gelatinase-associated lipocalin
